# Meeting Community Health Worker Needs for Maternal Health Care Service Delivery Using Appropriate Mobile Technologies in Ethiopia 

**DOI:** 10.1371/journal.pone.0077563

**Published:** 2013-10-29

**Authors:** Alex Little, Araya Medhanyie, Henock Yebyo, Mark Spigt, Geert-Jan Dinant, Roman Blanco

**Affiliations:** 1 Digital Campus, Winchester, United Kingdom; 2 Department of Surgery, College of Health Sciences, University of Alcalá, Madrid, Spain; 3 Department of Public Health, Mekelle University, Mekelle, Ethiopia; 4 CAPHRI. School for Public Health and Primary Care, Maastricht University, Maastricht, Netherlands; University of Alabama at Birmingham, United States of America

## Abstract

**Background:**

Mobile health applications are complex interventions that essentially require changes to the behavior of health care professionals who will use them and changes to systems or processes in delivery of care. Our aim has been to meet the technical needs of Health Extension Workers (HEWs) and midwives for maternal health using appropriate mobile technologies tools.

**Methods:**

We have developed and evaluated a set of appropriate smartphone health applications using open source components, including a local language adapted data collection tool, health worker and manager user-friendly dashboard analytics and maternal-newborn protocols. This is an eighteen month follow-up of an ongoing observational research study in the northern of Ethiopia involving two districts, twenty HEWs, and twelve midwives.

**Results:**

Most health workers rapidly learned how to use and became comfortable with the touch screen devices so only limited technical support was needed. Unrestricted use of smartphones generated a strong sense of ownership and empowerment among the health workers. Ownership of the phones was a strong motivator for the health workers, who recognised the value and usefulness of the devices, so took care to look after them. A low level of smartphones breakage (8.3%,3 from 36) and loss (2.7%) were reported. Each health worker made an average of 160 mins of voice calls and downloaded 27Mb of data per month, however, we found very low usage of short message service (less than 3 per month).

**Conclusions:**

Although it is too early to show a direct link between mobile technologies and health outcomes, mobile technologies allow health managers to more quickly and reliably have access to data which can help identify where there issues in the service delivery. Achieving a strong sense of ownership and empowerment among health workers is a prerequisite for a successful introduction of any mobile health program.

## Introduction

There is considerable enthusiasm for mobile health (mHealth) interventions and it has been argued that there is huge potential for mobile-health interventions to have beneficial effects on health and health service delivery processes, especially in resource-poor settings [1,2]. While a number of innovative mHealth projects have been launched in Ethiopia and other low-income countries in the past years, many have been short-term or have covered a limited geography [3,4]. Recently, the Ethiopian Federal Ministry of Health (FMoH) has identified the need to develop a scalable and comprehensive mHealth platform and strategy that could meet long-term needs and strengthen the primary health care system [5,6]. 

mHealth is a term used for interventions and programs designed to support medical and public health through the use of mobile technology [7,8]. The term commonly refers to mobile communication devices, such as mobile phones and smartphones, to deliver health services and transmit health-related information. mHealth ranges from simple mobile-based phone applications for the transfer of health information on basic handsets via short message service (SMS) to highly sophisticated diagnostic applications that rely on more advanced equipment (smartphones and tablets) and robust back-end data systems [9,10,11]. The 2011 mHealth in Ethiopia report identified five priority areas where mHealth could best help to strengthen the primary health care system: referrals, data exchange, supply chain management, training, education and consultation [5]. 

Ethiopia’s health needs are vast and reflect the high poverty levels, with more than thirty million people living in extreme poverty. In the last decade, real improvements have been seen in health services and outcomes, including a significant reduction of under five mortality, but this was from a very low baseline and huge challenges remain to guarantee Ethiopians have access to quality maternal and newborn health services [12]. Around 90% of all births take place at home and only 26% of women living in rural areas who give birth receive antenatal care from a skilled health provider and less than 3% of women receive postnatal care in the first week after delivery. Despite recent advances, neonatal mortality rate is still 37 deaths per 1000 live births and 1 in 17 Ethiopian children dies before their first birthday [13].

Ethiopia’s Health Extension Program (HEP) is a key component of the strategy to address these reproductive, maternal and newborn health program barriers. Strengthening and supporting the HEP is crucial for further acceleration of progress towards health-related Millennium Development Goals [14,15,16]. This is the primary channel through which health education, basic curative care and preventive components of primary health care reach Ethiopia’s population. HEP’s primary implementers are Health Extension Workers (HEWs) who as the frontline workers in the country’s health system interact with communities and families. HEWs have a variety of information and communication needs, and their ability to effectively communicate and exchange information directly impacts their ability to provide care to the communities they serve [17,18,19]. 

Whilst the Ethiopian FMoH is eager to explore the use of mobile technologies, without solid evidence of the health benefits and cost-effectiveness, they are reluctant to invest the scarce resources in widespread implementation [20,21,22,23,24].

Our aim in this study has been to meet the technical needs of Health Extension Workers (HEWs) and midwives for maternal health using appropriate mobile technologies tools. The health workers in our maternal health care project have now been using smartphones for more than twenty months, so we have been able to build up a good picture about what works and where there are issues. Most of the information in this paper is based on field reports our research team at Mekelle University (AM, HY) have been sending back following the training sessions they have been running and followed up discussions with the health workers.

## Materials and Methods

### Background: setting

This study was conducted in Tigray region, the northern most regional state of Ethiopia. Two districts,Kilte Awelalo and Hintalo Wajerat, were selected for the study, in consultation with the regional health bureau. In total, taking into account staff replacements, 20 HEWs, 12 midwives and 5 supervisors were involved. Equal proportions of health workers, health posts and health centers were selected from each district. Access to transportation and GPRS network coverage were the two main criteria considered for selection districts and health facilities. The study was approved by the health research and ethics review committee at the College of Health Sciences of Mekelle University. Training of the health workers and deployment of the platform began in August 2011. The data in this paper includes all the real patient encounters recorded between December 2011 and May 2013.

### Ethics statement

The study was approved by the health research and ethics review committee of the College of Health Sciences of Mekelle University (no: ERC 0032/2011). Written consent for participation was obtained for each health worker. The health workers were informed about their right to withdraw from the study at any time.

### Development: Case management tools and scorecard/analytics dashboard mobile applications

The technical components developed and deployed as part of this project cover: 1) case management tools; 2) Scorecard**/**analytics dashboard

These components have been built on systems already available, using open source components as far as it’s been possible. Any new code and content developed through this project has been released under the appropriate open source or creative commons license models. The technical development methodology was rapid, agile and iterative, allowing us to respond very quickly to changes in users needs/demands and in response to user feedback. 

#### Case management tools

We used the OpenDataKit platform (ODK) for the case management tools development [Appendices 1 and 2]. ODK is a generic data collection tool, designed for workers in the field, especially those with poor or no mobile internet connectivity, to collect data which can then be submitted to a central server when a data connection becomes available. The decision to use ODK was based on several factors: a) open source - so we were able to implement localisations and customisations and host our own back-end server to directly access the database, required for developing the scorecard and analytics dashboard; b) supports XForms standard - and so supports a wide variety of question types - text/numeric entry, multiple choice and multiple select and displays these to users on mobile devices running Android. In addition, it can handle Global Positioning System (GPS) location information, photos, videos, audio, and barcodes; c) flexible to expand for other areas, such as basic stock control, immunisation records.

Several customisations were required to make the phones and ODK suitable for use with the health workers in Ethiopia: a) local language support; b) supporting local calendar and c) ODK widgets.

**Figure 1:**
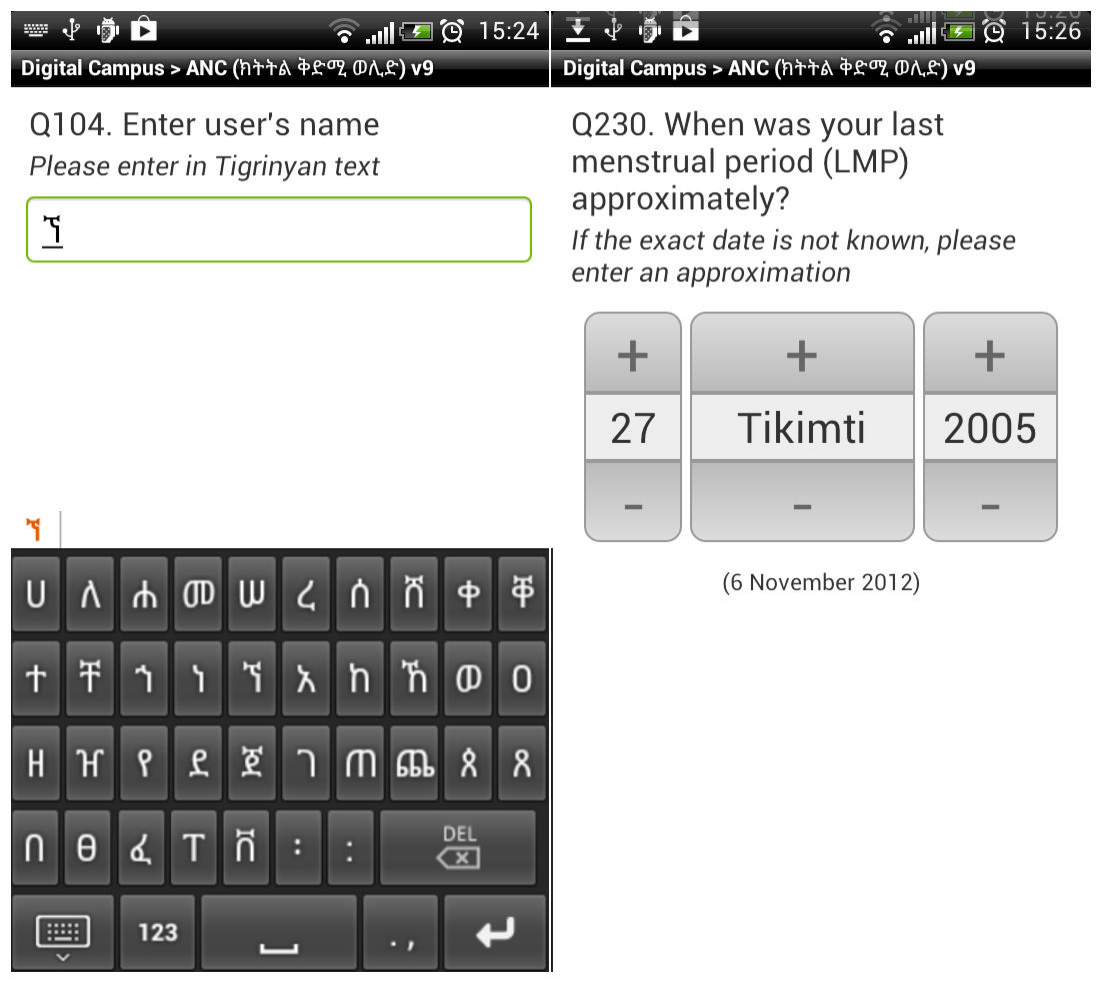
1a & 1b. Ge’ez keyboard and Ethiopian data picker.

The native language for all the HEWs, midwives and supervisors is Tigrinya, which uses a non-Latin script, Ge’ez. The version of Android we were using (version 2.3) does not natively support the Ge’ez script, although this script is supported in more recent versions of Android (version 4+). We installed an additional system font on all the phones to allow displaying text in Ge’ez, installation of this also required all the phones to be rooted. To allow data entry in Tigrinya or Amharic we developed a Ge’ez virtual keyboard, since at the time, no Ge’ez capable keyboards were available for Android, although now other Ge’ez keyboards are available. This allowed the health workers to decide for themselves which keyboard they preferred to use and whether they preferred to enter text data in either Ge’ez or Latin scripts (Figure 1a and 1b).

Ethiopia has its own calendar based on the Julian calendar, which is widely used in preference to the Gregorian calendar, especially in rural areas. We developed an Ethiopian date widget for ODK to allow health workers to enter dates in the calendar system they understood well. In addition to the Ethiopian date widget, we also developed three other ODK widgets to assist health workers in scheduling. One for calculating and displaying the expected delivery date (EDD), based on the last menstrual period (LMP) entered and two more widgets for giving suggested next antenatal care (ANC) and postnatal care (PNC) appointment dates, again based on the LMP date entered. For the appointment dates, only a suggested date range was given, the health workers and patients were free to enter whatever date may be most suitable for them. The pregnancy calculator was also transformed into a standalone Ethiopian Pregnancy Calculator application.

#### Scorecard and analytics dashboard

We developed an analytics dashboard and a mobile scorecard to allow HEWs, midwives, their supervisors and the local health bureaus to track the progress of pregnant mothers, their medical and pregnancy risk factors, and a range of key performance indicators. Providing information back to health workers and their supervisors about their performance, was designed to help the health workers manage their workload and patients. Performance indicators included the number of ANC, Delivery and PNC visits made.

ODK is primarily a data collection tool and it has no built-in functionality for creating customized reports back to mobile users. We built both the analytics dashboard and the mobile scorecard to access the ODK database directly to provide customised reports and information back to health workers, supervisors, local health bureaus and the research team. Although the analytics dashboard and the mobile scorecard both derive the data from the ODK database, each were created for different use cases and targeted towards different user groups, although any registered user may access either should they wish. 

We felt it was important that the data collected by health workers could be used directly by them to assist them in their work, for example scheduling appointments, risk factor assessment, and for them to feel ownership of the project and data, rather than a health management information system, used only by health bureaus and the FMoH.

#### Analytics dashboard for local health bureaus management and the research team

**Figure 2:**
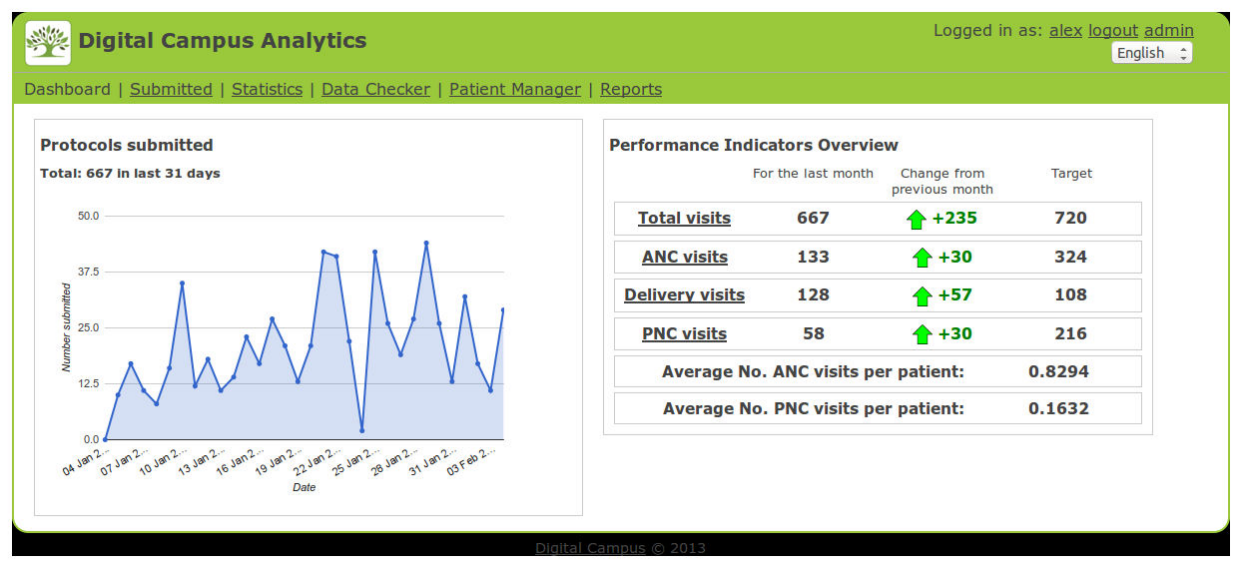
Analytics Scorecard home page.

These groups would be most interested in getting an overall picture of the level of activity in different health posts and centres, key performance indicators and the ability to compare performance between different health posts, centres or districts. Regular reports were available for supervisors and health bureaus, as well as full details of any protocol forms entered so they could be printed as a paper backup or reference at the health post (Figure 2). 

The analytics dashboard also highlighted inconsistencies in the data, for example where a patient id may have been used twice for different patients. We made the assumption that these users would have access to a laptop or personal computer (PC) with a reasonable internet connection, so the analytics dashboard is web based and designed to run in a full laptop or PC web browser, with an active internet connection.

#### Mobile scorecard for the HEWs, midwives and supervisors

**Figure 3:**
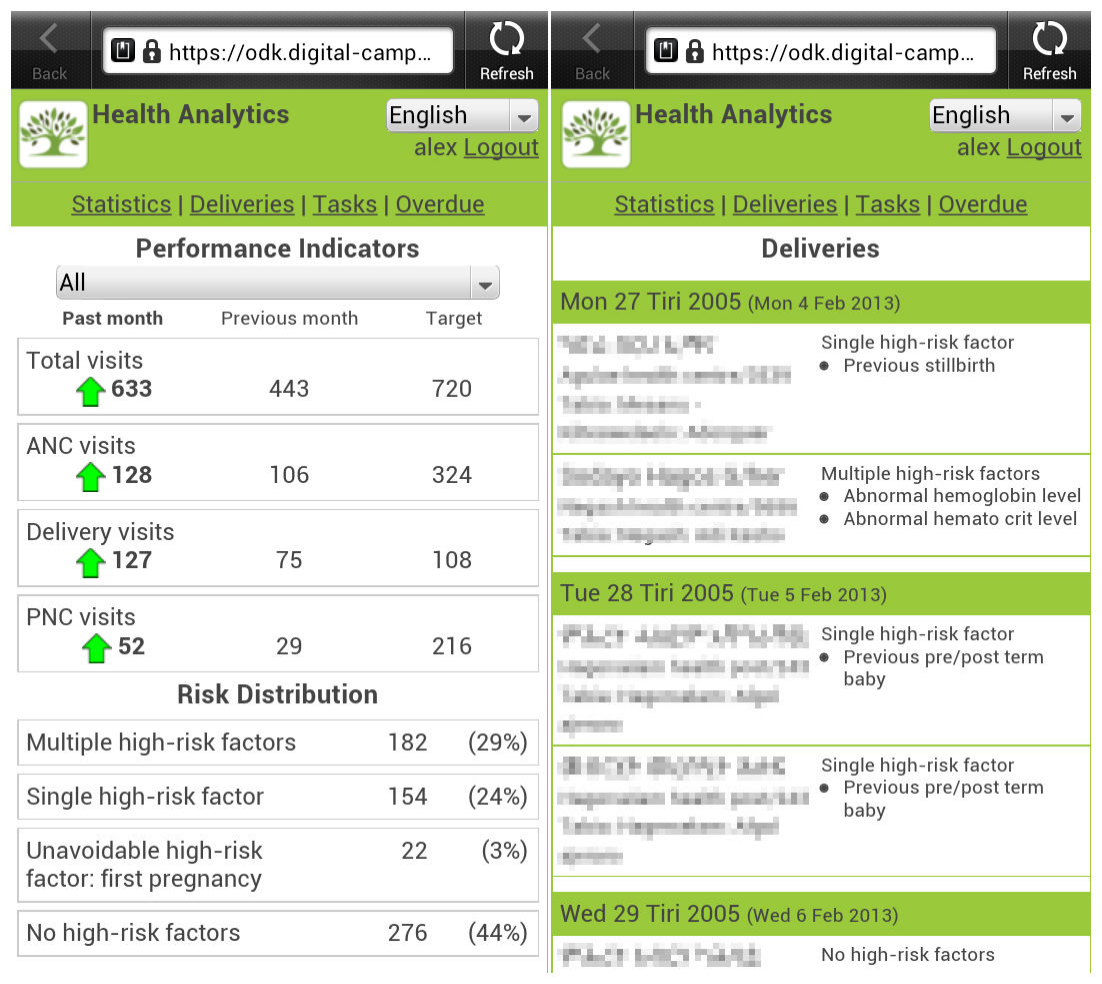
3a & 3b. Mobile scorecard homepage and mobile scorecard. Showing deliveries due and associated risk factors (personal data has been pixelated).

These staff, based in more rural areas, require information such as which pregnant mothers are due for maternal care (ANC or PNC) visits or delivery in the coming days/weeks, and any associated risk factors. Midwives at local health centres need information about upcoming deliveries in their district, so any special preparations can be made for delivery, especially with high risk cases, even when they may not have seen these patients before (Figure 3a&b).

Access to this data was provided by an Hypertext Markup Language (HTML5) web application, which could be accessed via the web browser of their smartphone. Using a local database for the web app, information would be cached and accessible even when they had no internet connection. The data would update whenever an active connection was available. Four key areas of information were provided in this application: a) performance indicators - number of different visits (ANC, PNC and delivery) made in the last month compared to the previous month; b) Deliveries due in the next month – with basic patient information (name, identification (ID), village and phone number if available), and risk factor analysis; c) Tasks and appointments due in the next month - basic patient information (name, ID, village and phone number if available) and type of appointment; d) Appointments overdue (missed) - basic patient information (name, id, village and phone number if available) and type of appointment. 

Given the reliability and speed of the mobile internet connection available to these health workers, we kept the amount of information cached on the phone to the minimum. Neither the analytics dashboard, or the mobile scorecard gave users any access to edit or change any data.

Details to obtain all the code for the technical development, as well as access to the demonstration site, is available in appendices S1 and S2.

### Implementation, Monitoring and Evaluation

#### Monitoring and evaluation: data analysis, follow up, user survey

During data collection period, monthly visits were made to the participant health posts and centres. Telephone contact was used often, especially when any problems with the phone usage and protocols registration were detected in the analytics dashboard and required consultation with the health worker. A mixed method of both qualitative and quantitative approaches was used to explore the feasibility of implementing these mHealth tools. In-depth interviews took place in the middle of the actual implementation period. It was semi-structured with open ended and probing questions. All interviews were tape recorded and all data was transcribed verbatim in the local language and then translated into English. Respondents were asked to evaluate the reliability of the solution as well as their satisfaction. Detailed results of this survey are being explained in another submitted manuscript.

#### Training for Health Workers

We pre-tested the customized ODK application and maternal health care protocols and trained health workers over a period of three months, with a mix of group and outreach training. The training included basic functions of the smartphone, configuring GPRS internet connection on the smartphone, purpose and content of the maternal health care protocols, installing and launching ODK software, switching from English language to local language and vice versa. During this pre-test phase, health workers practiced with the smartphone, and customized ODK, and maternal health protocols by submitting practice data to the server. During each training day, feedback was collected and improvements on the protocols and application were made accordingly. Actual implementation of the whole package of the application and assessment of its feasibility at the health posts and health centers was carried out from December 2011 to May 2013. Health workers were using the smartphones when giving services of ANC, delivery and PNC to women and were submitting real data to the server. During this phase, onsite supervision was made every month by the research team.

#### Protocol and form development

Initial versions of the protocol forms were developed by the research team, these were then iteratively refined based on feedback from health workers during the pre-implementation phase (Table 1). Due to the complexity/length of these protocol forms, most of the editing was done in an XML editor, rather than one of the automatic tools for building the forms. Once the protocols were finalised, all the text was translated into Tigrinya, so the health workers had the option to view the questions in English or Tigrinya. 

**Table 1:** Full list of maternal and neonatal protocol forms used.

**Protocol**	**Comments**
Registration	Initial registration of the patient
ANC First Visit	The first antenatal care encounter
ANC Transfer	For patients who receive care at more than one health post/centre
ANC Follow Up	Follow up antenatal care encounters
ANC	This was introduced in may 2012 and replaced the ANC First Visit, ANC Transfer and ANC Follow Up protocol forms
ANC Lab Test	Lab test results when patient visited the health centre
Delivery:	Delivery/labor
Termination	For recording premature termination of pregnancies (e.g. induced or spontaneous abortion)

The full forms are available at: https://github.com/alexlittle/Digital-Campus-Protocols.

#### Data security

To maintain security of the medical information transmitted between the phones and the server, all connections between the phone and the server were made over secure http using an SSL certificate. Once data entered in ODK was submitted to the server, the data was no longer stored on the phones and users needed their username and password to access either the mobile scorecard or analytics dashboard. Once logged into either of these, users are only able to view information related to patients in their districts. Full patient records were not stored on the phone. 

#### Patient identification

Patient identification (patient ID) was an important issue, since there isn’t a standard regional/national identification number we could readily use. Each patient encounter is recorded in a physical log book and the patient ID was simply the number of the next row in their log book. To try to save confusion between patients having different patient IDs in the log book and the electronic protocols, we identified patients by a combination of a health post code number (selected as the health post/centre name when viewed by health workers) and the id from the log book. Patients were given a card with their details to present when they returned for another appointment, whether at the same health post/centre or a different one. In addition to the health post and patient id, every protocol asked for the patients year of birth, age and first name - so we could use this information to determine if a patient id number may have been incorrectly input. For reference, in the rural areas many people do not know their exact date of birth and hence age, making it difficult to use these as reliable aids for patient identification, however we could use these to highlight where there may be a discrepancy.

#### Technical support

Day-to-day technical support was provided by the local research team, including installing the phone system, applications, protocol forms and dealing with any queries from the health workers. Any issues the local research team couldn’t resolve were passed to the Digital Campus technical team for investigation.

#### Phone battery recharging

Since many of the health workers did not have reliable access to mains electricity supply, all were provided with a solar lamp and phone charger. We originally provided a d.light (San Francisco, USA) but later changed to supplying ST2 solar lamp/chargers from the Solar Energy Foundation (Addis Ababa, Ethiopia), since these were available for purchase and supported in country. During the training sessions health workers were shown how to turn off/on the Wifi / Bluetooth / GPRS / GPS to help improve battery life.

#### Phone usage

We placed no restrictions on the phones regarding the applications which could be accessed or what the top-up balance could be used for. The health workers were free to use and install any application, including using the phone, text-messaging and internet browser. Each health worker was provided with a 100 birr (five point three USD) top up card approximately once a month, and they were free to purchase and use additional top-up cards. We were able to obtain phone usage information - amount of top-ups and how much spent on voice, SMS and data each month - directly from the local mobile operator, EthioTelecom.

### Technical specifications

#### Smartphones

HTC Hero smartphones were purchased second hand, in order to keep the project costs down. Phones were unlocked, rooted and Cyanogen, a custom operating system distribution was installed. Each health worker was provided with a charger/adapter, cables, solar lamp/charger, plus an SD card of at least 2Gb. Dual battery chargers and extra batteries were also provided for those who had very poor mains electricity access, or who often spent extended time out of their health posts. With every phone we also gave a rubber or plastic protective cover and a small bag with shoulder strap for protection.

#### Server/Software

The server (Dell PowerEdge) is running as a Ubuntu 10.04 (LTS) virtual machine (using VirtualBox), with MySQL 5, PHP 5, Apache 2 and Tomcat 6. The ODK software, both ODK Aggregate and ODK Collect has been kept up to date with current stable release versions, currently running ODK Aggregate 1.0.4 and ODK Collect 1.1.7. 

## Results

The key results have been separated into four categories below. Overall we found fewer technical issues than initially expected and the health workers very quickly became comfortable in using the phones.

### Technical - hardware/infrastructure

The mobile internet connection, although not fast, was found to be much better than originally expected, even in rural and quite remote areas. 23 health posts and centers from a total of 47 (48.9%) had GPRS connection available at the time we visited (April 2011). Only those who were in an area of connectivity during this survey were included in the project. We had very few instances of the mobile data network being unavailable for a substantial period of time (more than one day). Contact with the local telecoms office informed us of the nature of the problems and when they were likely to be resolved. In April 2013 GPRS connection was available in 35 health posts and centres (74.4%) of our study districts. We had very few instances of the mobile data network being unavailable for a substantial period of time (more than one day). Contact with the local telecoms office informed us of the nature of the problems and when they were likely to be resolved.

Our initial expectation was that we may need to replace 25% of the phones through loss or breakage, according to previous reports. Until May 2013, only two phones out of 36 had been stolen - but one was later recovered (2.7%). Three phones (8.1%) had issues with insensitive screens and were replaced. This low level of breakage/loss was very significant, especially since we were using second-hand phones.

The phone model (HTC Hero) chosen for this study had an initial cost at eBay.com of one hundred and sixty-nine USD for lightly used models while retail value was five hundred and thirteen USD for new ones (2010). The cost of the phones was reduced more than 50% in the in the following months (down to 75 USD by January 2013).

**Figure 4:**
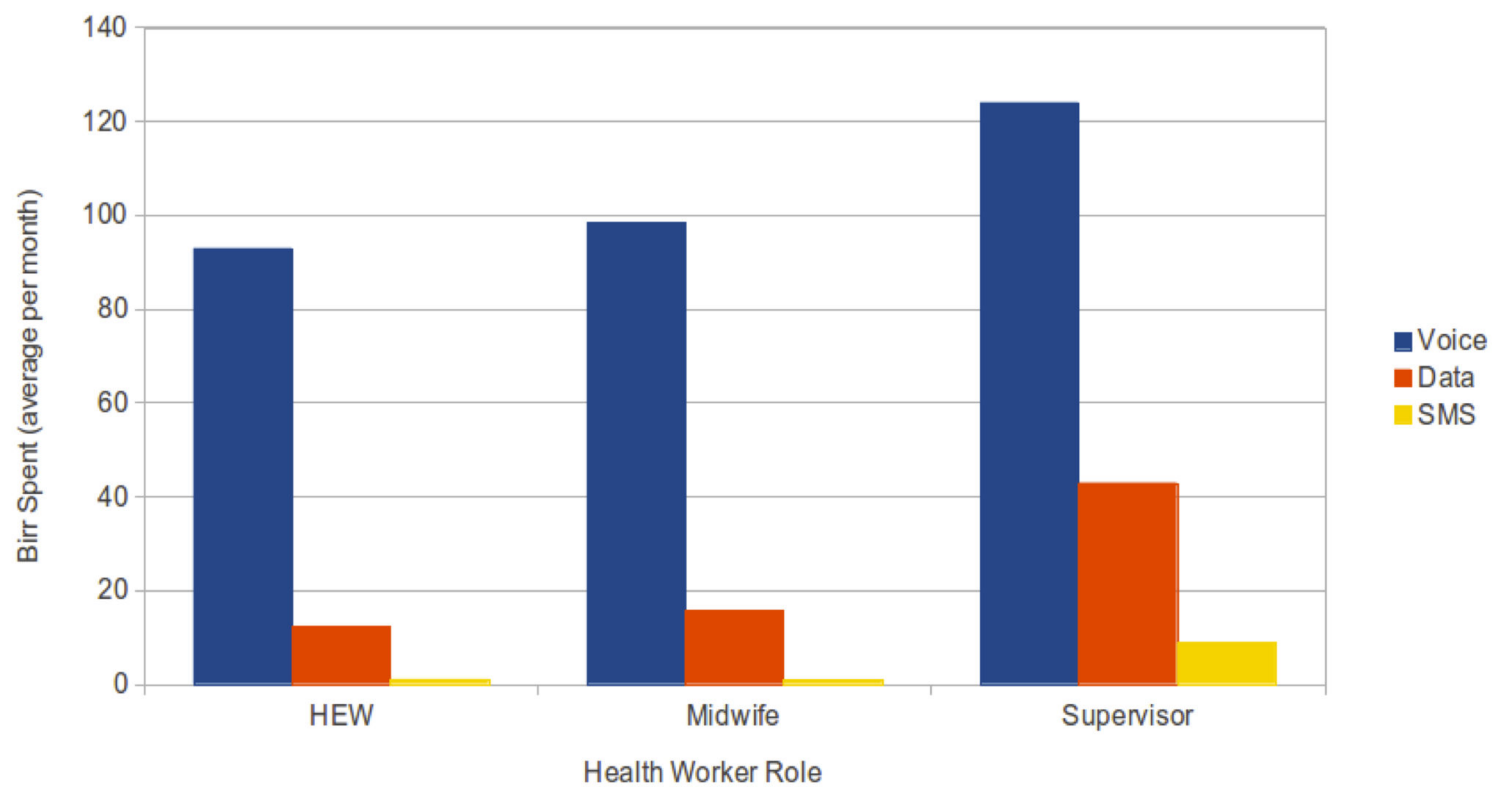
Amount spent per health worker on voice, data and sms per month. Graph of the average monthly expenditure of the health workers on mobile top-up cards broken down by money spent on voice, data and SMS. Interestingly, the number of SMSs sent by HEWs and midwives was very low.

Figure 4 shows a graph of the average monthly expenditure of the HEWs, midwives and supervisors on mobile top-up cards broken down by money spent on voice, data and SMS. Table 2 shows how this money spent translates into minutes of voice calls, data downloaded and SMSs sent per month. The number of SMSs sent by HEWs and midwives is very low. One possible reason for this - and this also came from our baseline survey interviews - was that the health workers don’t use text messaging because they are not confident in using the Latin alphabet, or perhaps they know the recipient of the message cannot read the Latin alphabet, or does not have a Ge’ez capable phone. From the amount of data usage, we can see that both health workers and supervisors are using the data connection for more than just submitting patient encounter records and the mobile scorecard and in the case of supervisors, substantially more. The data shows that each health worker per month makes approximately 160 mins of voice calls, downloads 27Mb of data and sends 3 SMSs. The health workers were adding their own top-up balance too in addition to the five point three USD we were giving. What was interesting for us is that the health workers are clearly using the data connection for much more than simply submitting the protocol forms and accessing the mobile scorecard.

**Table 2:** Average money spent translated into minutes of voice calls, data downloaded and SMSs sent per month.

**Role**	**Voice calls (min)**	**Data downloaded (Mb)**	**SMSs sent**
HEW	157.4	24.4	3.0
Midwife	166.8	31.5	2.6
Supervisor	210.1	85.8	25.1

### Technical - software

Input from the health workers was critical in the development of the protocol forms, which questions should be asked and clarifying any misunderstanding and ambiguities in the questions and response options. After producing an initial draft of the protocols, we made several revisions of each protocol form and its questions, based on input from the health workers and analysis of how they were using the forms for practice data entry. These iterations allowed us to identify potential problems before starting to collect data on real patients. 

In using ODK for gathering information over an extended period of time, with different forms completed at different times, we are essentially forcing ODK to do something it was not designed for. Although ODK assigns a unique identification number to each form submitted, there is not a built-in way to link different form instances, either of the same or different form types, submitted at different times, which is required for gathering longitudinal information, hence we used the patient id to match up forms. Since the patient records were not permanently on the phones, we were depending on the patient ID being entered correctly to match up different encounters with a single patient. Patient id issues are discussed below.

We found that occasionally health workers would adjust the phone system date to match the Ethiopian calendar. For example, setting the phone date to 2 September 2005, to represent the date 2 Meskerem 2005 in the Ethiopian calendar, but which actually corresponds to 12 September 2012 in the Gregorian calendar. This led to the health workers being unable to submit protocols to the server because the phone operating system assumed the security certificate was not within it's validity period and their connections to the server were rejected.

In the early phases of the project, when the protocol forms were being refined and updated, we needed a well coordinated approach to transfer data and users from using one version of a particular protocol form to the updated one. ODK currently does not have functionality to automatically push out updates to the users’ phones and transfer all users to the new protocols at the same time.

Although we did not restrict the phone usage in any way, health workers had access to to change any and all of the system settings, install/remove applications etc, we had a very low number of issues. Almost all the support issues we did experience (for example, the ODK application accidentally being uninstalled) were easily managed by the local research team.

### Usage Statistics, Data Quality and Consistency

**Figure 5:**
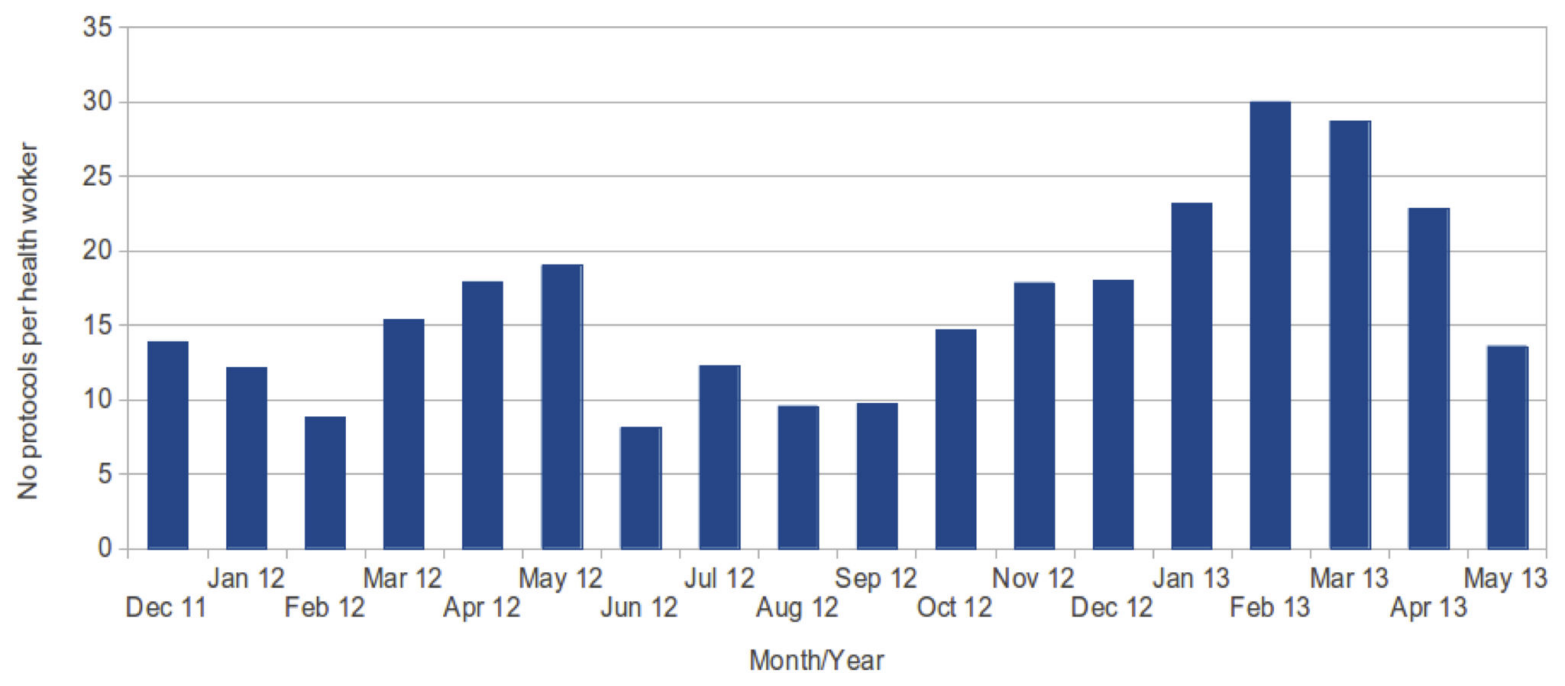
Average number of protocol forms submitted each month (per health worker).

From figure 5 there appears to be no evidence of increased usage of protocol forms by health workers over time. However, given the only incentive to use the application was to assist them in their work, and there was no penalty if they did not use the application, it is encouraging to see that there is continued usage and no drop in activity.

**Figure 6:**
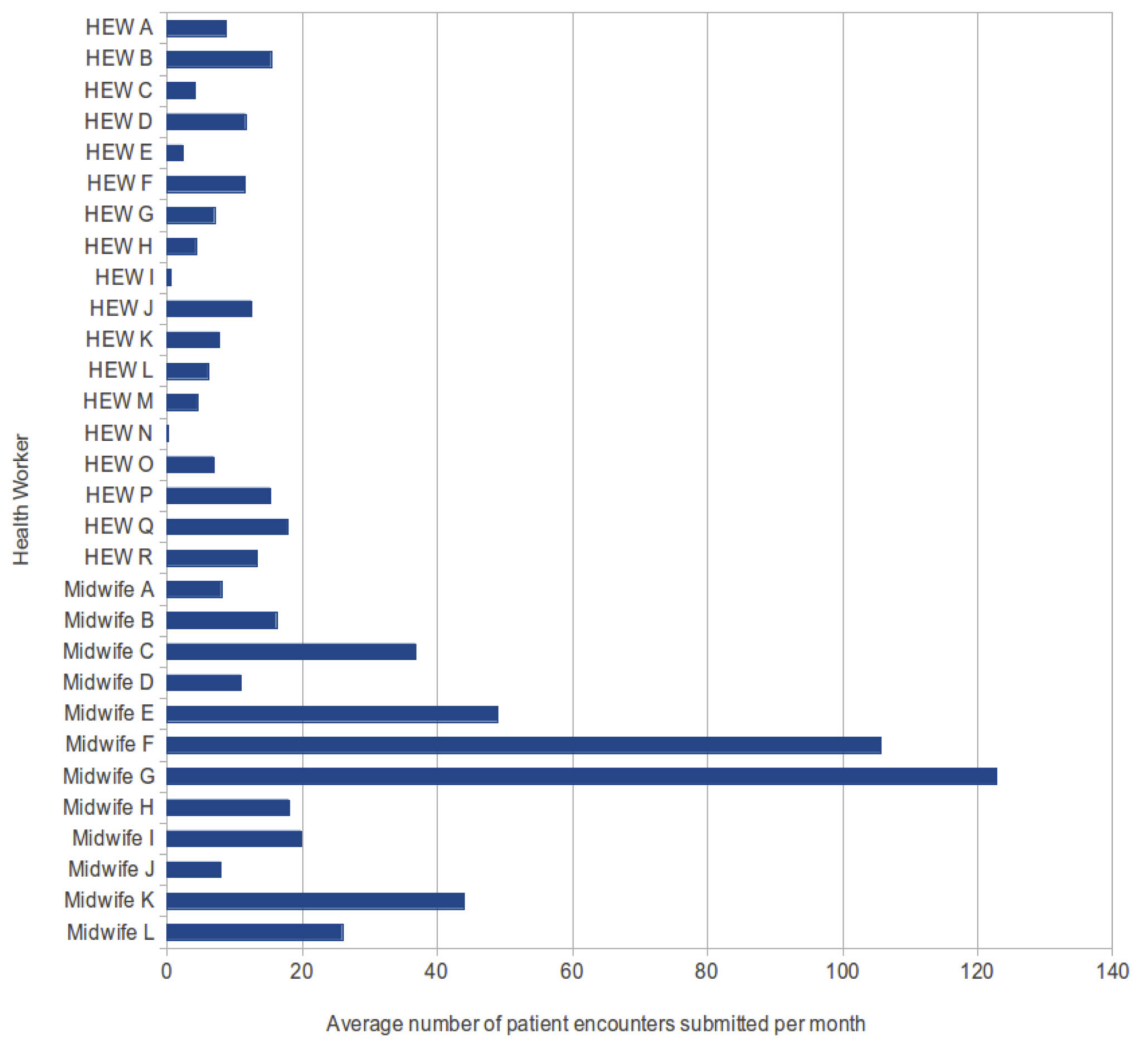
Average number of protocol forms submitted per month by individual health workers (HEWs and midwives).

**Figure 7:**
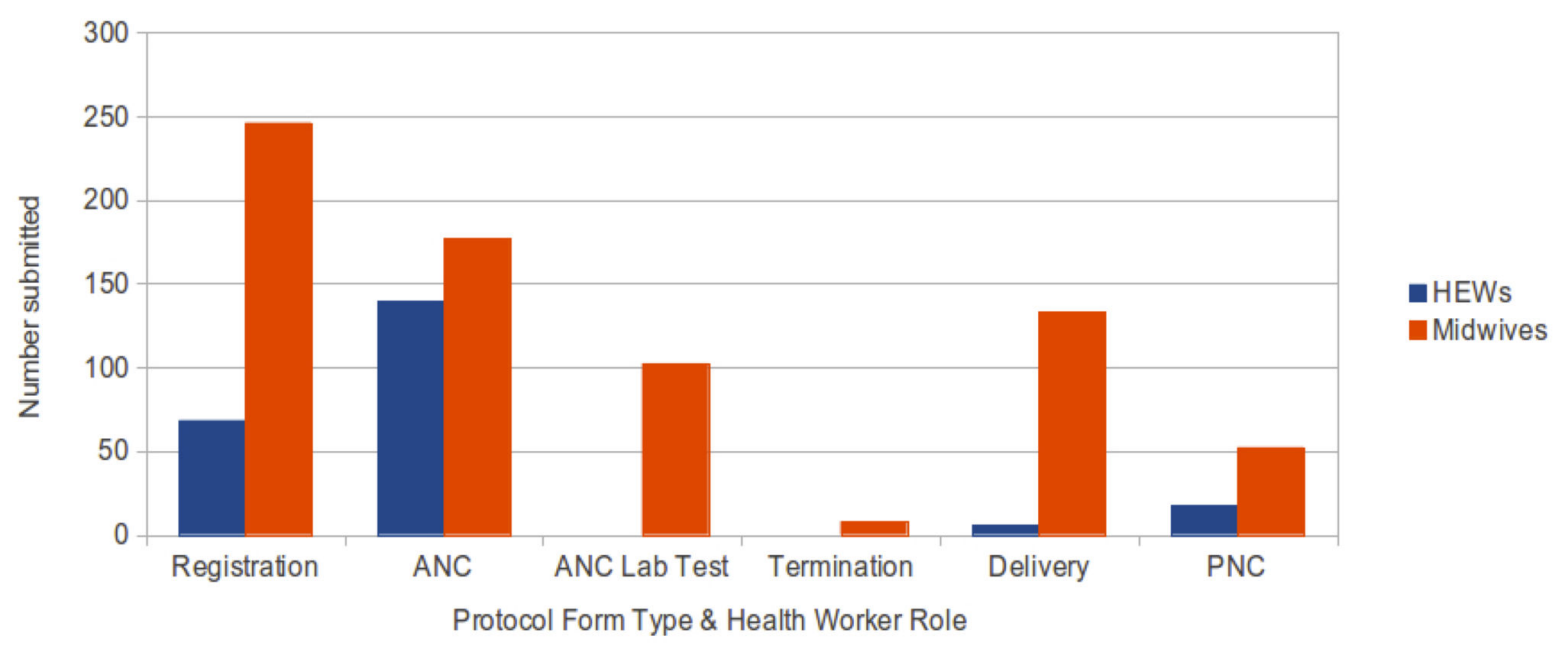
Sum of the monthly averages by HEWs and midwives for each protocol type. The difference between the rate of ANC and PNC protocols, especially for HEWs, probably reflects the priority which has been given to antenatal care compared to postnatal care and shows there is a lot of room for improvement in postnatal care provision.

From figure 6, we can see that the midwives were substantially more active in submitting all types of protocols than the HEWs, although we would not expect HEWs to enter the ANC Lab Test protocol because these tests are always done by midwives, nor a high rate of delivery by HEWs since current Regional Health Bureau advice is that HEWs should not routinely assist with delivery. The difference between the rate of ANC and PNC protocols, especially for HEWs, probably reflects the priority which has been given to antenatal care compared to postnatal care and shows there is a lot of room for improvement in postnatal care provision (Figure 7). The usage of each type of protocol and the number of ANC and PNC encounters per health worker is in line with data from the EDHS [13]. In addition to the difference in activity between different health worker roles we noticed highly variable usage between different health workers and between different health posts and centres. Discussion of these issues is beyond the scope of this technical paper and it is being developed in another manuscript. 

**Figure 8:**
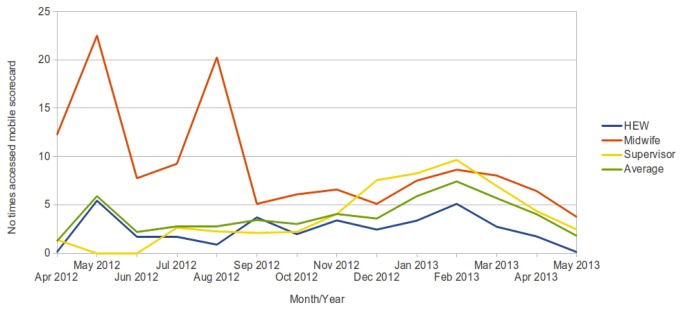
Average number of accesses to the mobile scorecard, by health worker role.

As with the number of protocol forms submitted, we see varying levels of activity between different health posts/centres and roles (Figure 8). Little input from supervisors as to exactly what data they needed to help improve their supervisory role was obtained. We were given various statistical reporting template forms from local health bureaus, and we could have used data from our database to help complete this information. However, the reports seemed to vary significantly from area to area and the indicators reported on regularly changed.

Although the health workers all seemed to well understand the system for patient identification, we had many issues where the same patient ID was used for multiple patients. We identified approximately 6% of all protocol forms entered had an issue regarding patient identification. The check data used in each protocol (name, year of birth and age) helped us to determine where a patient id may have been used more than once or duplicate protocol forms being submitted. It should be noted there is naturally significant overlap between duplicate patient ids and inconsistencies in the age/year of birth. Possible causes for duplication of patient ids were the following:

1. The protocol is edited and saved as new protocol before submission, in which case 2 copies of the protocol form were submitted for the same patient;
2. Network connection issues - breaks in the network connection may mean the protocol form being successfully submitted to the server, but the notification of successfully submission is not received back on the phone, so the phone believes the protocol form has not yet been submitted, and so is submitted again;
3. Typo in the patient ID;
4. Restarting numbering in the log books - there appear to have been cases where the sequential numbering in the log book has restarted, for example at the beginning of a new year, meaning that patient ID number are reused for new patients.
5. Multiple registration systems. Some health facilities did not use a single registration system, patients were registered differently depending on what service they came to use, for example, one log book for terminations and another for deliveries, but both log books using the same sequence of patient ID numbers. This led to patients being given the same patient ID that had already been given to others.

Consequences of patient ids being entered incorrectly or duplicated include: a) Appointment reminders being allocated to the wrong patient; b) Risk factor analysis either highlighting more risk factors than the patient actually had or omitting risk factors for a given patient.

The analytics scorecard highlighted possible patient identification issues, and in theory the majority should have been straightforward to resolve, for example by contacting the health worker shortly after the error was made to establish what the correct data should be. However delays in looking into these issues meant that a large backlog of errors (going back several months) was time consuming to resolve. 

There may be instances where the same patient has been registered twice, for example, when attending a health post for an antenatal care encounters, and then re-registered at the health centre when attending for delivery. Variations in the spelling of names, especially when transliterated into the Latin alphabet and lack of consistent usage/knowledge of an exact date of birth, makes it hard to analyse, without significant effort, how many instances of re-registration occurred. 

**Figure 9:**
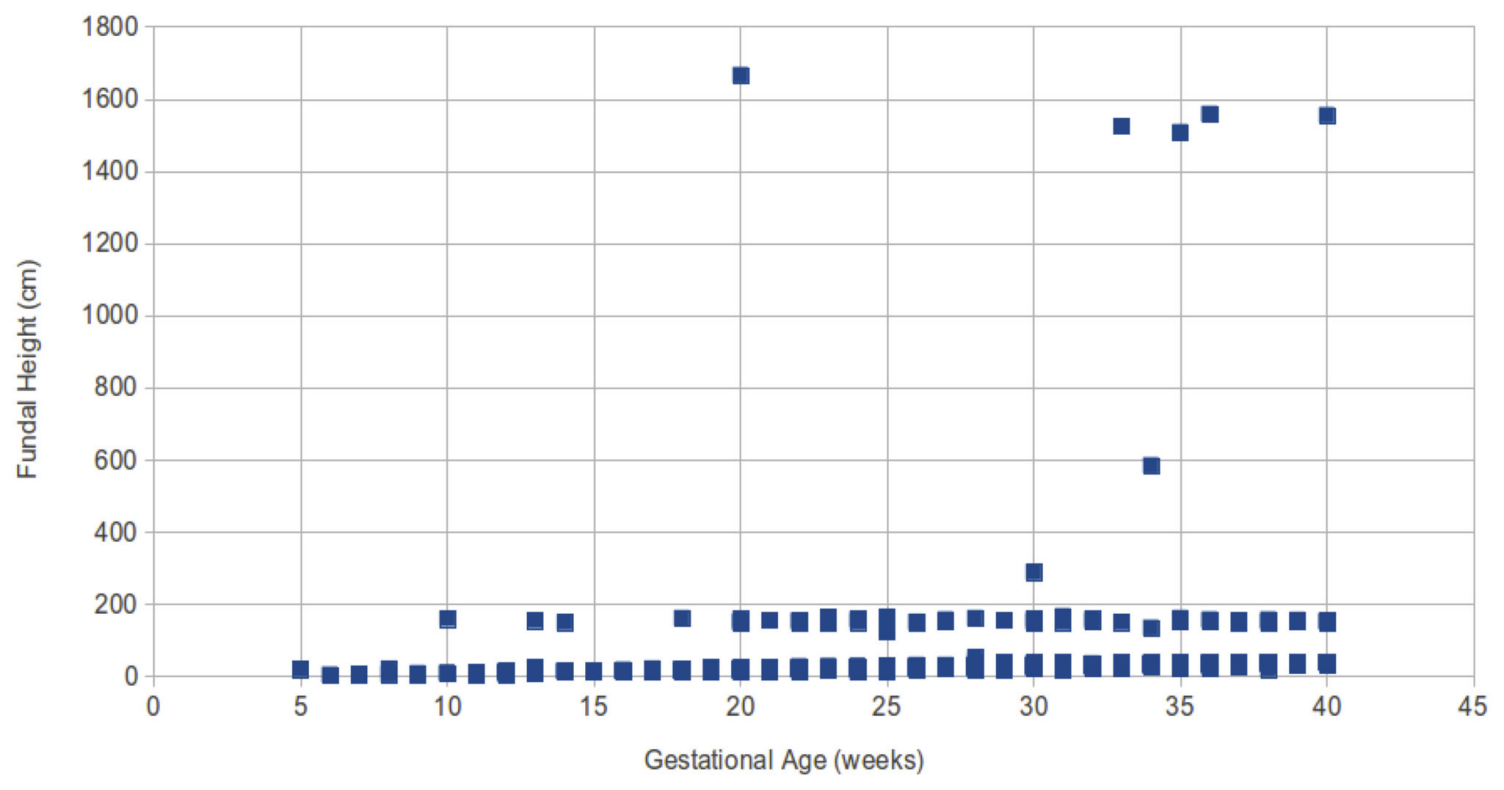
Scatter plot of gestational age against fundal height. The handful of data points on the top of the chart appear to show where typos have been made in data entry. However the second line data points around the fundal height of 150, appear to show a regular and consistent error being made.

The analytics dashboard allows us to identify where training issues may be the cause of incorrect data being entered. An example is the fundal height and newborn weight measurements. In figure 9 for the fundal height, the majority of the data entered is consistent with our expectations, with the data points along the very bottom of the scatter plot. The handful of data points on the top of the chart appear to show where typos have been made in data entry. However the second line data points around the fundal height of 150, appear to show a regular and consistent error being made. 

Analysis of the patient ages and parity shows we may have two underrepresented groups in our data, those who are pregnant for the first time and those who are under 20 years old. Discussions for the reasons for this are beyond the scope of this paper, but these findings highlight how the data collected could be used by health managers to ensure that health services are reaching those most at risk of complications during pregnancy.

### User Survey, Usability and User Acceptance

Most health workers in this project very rapidly learned how to use and became comfortable with the devices and the Android operating system, so after initial orientation to using the devices, only limited technical support was needed and most of this could be provided by the local support staff by phone or during periodic visits. The feedback we have received from the health workers has been overall very positive. 

Ownership of the phones was a strong motivator for the health workers, who recognised the value and usefulness of the devices, so took care to look after them. For most health workers the smartphones quickly became their main phone. We believe that this is because we trusted them with the devices and gave them the flexibility to use the devices for personal use too. Regarding personal usage, all the health workers had customised the phone background image using photos, usually of their family, and many had transferred music onto their phones. In addition to the top-up balance we gave them as part of the project, they also added their own balance to compensate for their personal usage of voice calls, SMS and internet usage.

Many health workers initially complained about the short battery life of the phones - especially when compared to their own standard mobile phones which may stay charged for several days to a week. Although we had given all the health workers solar lamp chargers and, in some cases, dual batteries, is seems few used this as the primary method for keeping the phones charged. Although less than 10% of the health posts were connected to mains electricity, most health workers did have access to mains electricity at home.

Not surprisingly, health workers mentioned that using the protocols took a long time. We knew that it was always likely that the protocol forms would increase the time for a patient encounter. Not necessarily solely due to the technology, but also because we were asking them to ask quite a comprehensive set of questions and a physical examination. Previously, without the electronic protocols, the patient encounters may not have been as thorough. From the start/end times, automatically logged by the phones, we could identify how long health workers spent for each patient encounter. For an ante-natal care first encounter the average time for the patient encounter was 20 minutes. In our visits, the health workers and mothers seemed very comfortable with using the protocols, as it checked that all the right questions were being asked during the patient encounter and the results are being published in a forthcoming paper.

The health workers seemed most comfortable using the Tigrinya versions of the protocols. Health workers could switch between English and Tigrinya and were free to enter text data in either Latin or Ge’ez script, although very few questions required any text input. They appreciated the flexibility allowing them to view and enter data in either English or Tigrinya and the fact they could use the Ethiopian calendar for entering dates.

Health workers appreciated that the changes made were based on their suggestions for improvements. They seemed keen to see us using the same system for other aspects of their work, for example integrated management of childhood illnesses, tuberculosis, immunisations and stock control. 

## Discussion

Introducing new technologies in environments such as primary health care in low-income countries will always be challenging. mHealth applications are complex interventions that require changes to the behavior of health care professionals who will use them and changes to systems or processes in delivery of care [25,26,26,27]. One of the key reasons that this project had significant activity levels and low device loss rate is because of the sense of ownership and intimacy the health workers developed to their smartphones. Because we did not restrict the usage of the mobile devices only to their professional work, the health workers appreciated how useful the smartphones can also be in their personal lives. Health workers were confident in experimenting with the devices, for example using the camera, loading music, accessing the internet and some have even created Facebook accounts - despite no training or advice on how to do this. For almost all the participants this was the first opportunity they have had to access the internet or use a powerful computing device [27,28,28,29]. 

We agree with other authors that in the context of limited resources, implementing untested mHealth interventions at scale without a proven theory of behaviour change is likely to result in many failed scale up projects and significant levels of wasted resources [26,27]. Because our health workers were able to develop this sense of ownership and empowerment, they were willing to take good care of the devices, for example, ensuring that they were always charged up, both with power and top-up credit. With the devices powered up with top-up balance and always to hand, and so always available for their professional work, rather than being left in a drawer somewhere, then not being charged up or with credit when they actually come to use them. They clearly developed a connection with and a motivation to use the phones that we may not see with other types of devices, e.g. a PC, and have no evidence that the health workers were abusing the lack of restrictions on personal use [20,29,30]. 

Inclusion of the health workers in the development of the protocols, as well as localisation into their local language (Tigrinya) and allowing dates to be entered and displayed in the Ethiopian calendar, also contributed to the sense of ownership. There is already good evidence that the use of standardised protocols can improve the accuracy and effectiveness of routine diagnosis and treatment, although there remains the need for developing a better mechanism of delivery of protocols to enable widespread use, and the use of mobile devices could bridge this gap [30,31,31,32,11]. 

The final aspect we felt increased the sense of ownership was the implementation of the mobile scorecard and a complete cycle of data. We wanted to ensure the health workers had a reason to enter the data, and they could see that it could help improve their professional work and managing their time for appointments, rather than purely collecting data on their performance, or for health management statistical information [32,33]. 

Our approach to unrestricted use varies somewhat from previous mHealth projects [31,32]. Trying to restrict usage of the device to purely professional, for example by locking down or uninstalling applications, could be a mistake in terms of getting engagement from the health workers. Similarly, we started from the point of view that we would provide the mobile devices. Relying on the low-end phones already owned by the health workers, we feel, would have severely restricted what was achievable, as we would always be working with the lowest common denominator of technology available. Since the health workers are already poorly paid, expecting them to provide their own devices for their professional work is expecting too much. In addition, other projects [33,34] have experienced issues with providing technical support when using the phones already owned by health workers and so had to change to providing devices.

The analytics dashboard was an extremely valuable tool for monitoring, giving us real time information on the encounters being made by the health workers. This allowed us to very quickly identify when there may be problems or issues, either with the mobile internet connection or the actual data entered. From the analytics dashboard data, we are able to identify a number of training issues. This has led us to develop a mobile learning application to assist in resolving these training issues. The mobile learning application uses content from the Ethiopian FMoH approved HEW upgrade training programme [HEAT reference, 35], supplemented with video and assessment exercises and can all be run offline on the phones. This application is under active development and we are in the process of trialling the application with a larger group of health workers. Further information about the mobile learning application can be found in appendix S3.

Although the usage level by some of the health workers was low (see Figure 6), especially compared to the midwives, possibly as a result of better training, higher professional status, higher wages, we can see that all the health workers quickly learn how to use the devices and protocol forms with a minimal amount of training, plus the technical infrastructure is good enough to run these types of systems. We could see that the health workers are able to enter data with a manageable amount of errors, as described [36], most of which could be resolved with a little extra training, supervision and practice. 

Regarding the software side of technical system, the ability to get a good overview in real time of the activity of the health workers was especially valuable. Given that ODK is not designed to support longitudinal information and the issues we have had regarding patient identification, some aspects of this technical implementation may not be suitable for scale up. Rather, a cut down version of an electronic medical records system (EMRS), a mobile lightweight EMRS, would perhaps be more appropriate, especially one which not only just stored a subset of patient records, but also some of the key performance indicators and assisted the health workers with managing appointments, risk factors, referrals and scheduling. 

Currently, it does not appear that a full EMRS system would either be required or could be successfully implemented. Development of open standards for all these systems is going to be particularly critical for equity in e- and mHealth. There is a need for a governing body to certify open standards and enable countries’ access to standards that meet criteria [26,25,37]. However, a full EMRS system [36,38] would not fix some of the underlying issues of the health system organization, such as improving the supervision of health workers, professional career development, staff turnover and the coordination and referrals between health posts and health centres.

Improved patient identification would be key to a very successful light-EMRS or full-EMRS. There are already some efforts in Ethiopia to resolve this identification issue, for example the national Health Management Information System or Family Folder system, but these aren’t fully rolled out to all the health posts we are working with, so we weren’t able to take advantage of these. A patient identification system which included using some form of check digit would seem particularly advantageous. Alternative biometric approaches, such as those currently being deployed in India [39], although valuable, may introduce technology dependencies and costs which may not be currently feasible in Ethiopia.

Nowadays, smartphones are still more expensive than standard mobile phones, and this has often been cited as a concern regarding scalability [32]. We would anticipate that cheaper and better devices matching the requirements described earlier would be available shortly. Using the phones for multiple applications, such as case management, stock control, mobile learning and assessment, will make them much more cost effective [27]. We have already started looking at which phone models may be a good replacement for the smartphones used in this project. In this regard, Android phones are beginning to be assembled in Ethiopia, so these could be a good alternative option to importing phones. The key factors in the decision of which mobile device to be used would be the following: 1) value for money; 2) runs a recent version of Android platform; 3) the device can be rooted easily, if necessary, to enable the installation of local language fonts; 4) hard-wearing and robust; 5) screen size, especially for delivery of multimedia content.

With the number of health workers included in our project, it has been too early to measure any significant impact on health outcomes. The project and data collection is ongoing, so we will continue to monitor the data for any evidence of this. A key challenge for the mHealth field is the need for better and more evaluation and the next step is that high quality trials should be conducted to establish the effects of clinical diagnosis and management support such as protocols and decision support systems on clinical outcomes using mobile devices. The effects of such support on the management of different diseases and on objective disease outcomes should be evaluated.

## Supporting Information

Appendix S1 **Source Code.** Information and links for accessing all the source code for the applications and our customisations.
(DOCX)

Appendix S2 **Demonstration Site.** A demonstration site of the analytics dashboard and scorecard applications.
(DOCX)

Appendix S3 **Mobile Learning Application** How to access and install the mobile learning application (OppiaMobile).
(DOCX)
