# Correction: Meeting Community Health Worker Needs for Maternal Health Care Service Delivery Using Appropriate Mobile Technologies in Ethiopia 

**DOI:** 10.1371/annotation/fedf94d2-cf4e-494c-8828-85861ce282a5

**Published:** 2014-01-30

**Authors:** Alex Little, Araya Medhanyie, Henock Yebyo, Mark Spigt, Geert-Jan Dinant, Roman Blanco

The fifth sentence of the sixth paragraph of the Discussion section should have cited reference 25 instead of HEAT reference, 35.

The third sentence of the ninth paragraph of the Discussion section should have cited references 26, 27, and 37 instead of 26, 25, and 37.

A reference was omitted from the last sentence of the seventh paragraph of the Discussion section.

The sentence should read: We could see that the health workers are able to enter data with a manageable amount of errors, as described [35,36], most of which could be resolved with a little extra training, supervision and practice. 

