# Supplementary material for: Meeting Community Health Worker Needs for Maternal Health Care Service Delivery Using Appropriate Mobile Technologies in Ethiopia
Source: PLoS One. 2013 Oct 29;8(10):e77563. doi: 10.1371/journal.pone.0077563 (PMC3812262; doi:10.1371/journal.pone.0077563)
Supplement: Appendix S1 — Source Code. Information and links for accessing all the source code for the applications and our customisations. (DOCX) [file pone.0077563.s001.docx]

**Appendix S1: Code source**

The code for all these developments are available to download. All these are released under open source/creative commons licenses to [clone](http://help.github.com/fork-a-repo/) and develop further:

- Customised ODK: code: <http://code.google.com/r/alextlittle-dc-odk/> and complied version: <http://alexlittle.net/blog/download.php?file=dc-latest.apk>
- Analytics dashboard (including mobile scorecard): <https://github.com/DigitalCampus/Digital-Campus-Analytics>
- Protocol forms: <https://github.com/DigitalCampus/Digital-Campus-Protocols>
- Ethiopian Pregnancy Calculator: code: <https://github.com/DigitalCampus/Ethiopian-Pregnancy-Calculator> and app: <https://play.google.com/store/apps/details?id=org.digitalcampus.edd>
- Amharic/Ge'ez Keyboard: code: <https://github.com/DigitalCampus/Amharic-Keyboard> and installation instructions/support: <http://alexlittle.net/blog/2011/06/03/installing-geez-virtual-keyboard-on-android-devices/>
