# Supplementary material for: Meeting Community Health Worker Needs for Maternal Health Care Service Delivery Using Appropriate Mobile Technologies in Ethiopia
Source: PLoS One. 2013 Oct 29;8(10):e77563. doi: 10.1371/journal.pone.0077563 (PMC3812262; doi:10.1371/journal.pone.0077563)
Supplement: Appendix S2 — Demonstration Site. A demonstration site of the analytics dashboard and scorecard applications. (DOCX) [file pone.0077563.s002.docx]

**Appendix S2: Demonstration site**

A demonstration site of the analytics and mobile site for the patient management tools can be found at: <http://odk-demo.digital-campus.org/scorecard/> (username/password is demo/demo) and the mobile version is at: <http://odk-demo.digital-campus.org/scorecard/mobile> (same username /password). The demo user has supervisor privileges, so is able to see all the data entered, usually health workers logging in would only get to see the data directly related to their patients. If anyone would like to see the whole process, from entering the protocols on the smartphone, all the way through to seeing the cases on the analytics scorecard and mobile site, a demo ODK Aggregate server for submitting protocols has been designed. To set this up: [Download](http://alexlittle.net/blog/download.php?file=dc-latest.apk) and install on the mobilephone our version of ODK and Start the app and enter the following settings (go to menu > change settings):

- - Server: http://odk-demo.digital-campus.org/ODKAggregate (note that this is case sensitive)
  - Username: demo
  - Password: demo

Go to ‘get blank form’ – this should connect to the server and show all the available protocol forms – select and download the ones you would like to try out and Enter and submit a few protocols from your phone.
