# Supplementary material for: Meeting Community Health Worker Needs for Maternal Health Care Service Delivery Using Appropriate Mobile Technologies in Ethiopia
Source: PLoS One. 2013 Oct 29;8(10):e77563. doi: 10.1371/journal.pone.0077563 (PMC3812262; doi:10.1371/journal.pone.0077563)
Supplement: Appendix S3 — Mobile Learning Application How to access and install the mobile learning application (OppiaMobile). (DOCX) [file pone.0077563.s003.docx]

**Appendix S3: Mobile Learning Application**

For a video demo see: [http://oppia-mobile.org](http://digital-campus.org/heat-content-on-smartphone-and-tablet/) and for a technical overview of this development see: [http://oppia-mobile.org/developers](http://alexlittle.net/blog/2012/05/03/openlearn-content-on-mobiles/).The application is freely available on Google Play (from: <https://play.google.com/store/apps/details?id=org.digitalcampus.mobile.learning>) so can be installed on any Android device.
